# Comparison of the neuromuscular response to three different Turkish, semi-professional football training sessions typically used within the tactical periodization training model

**DOI:** 10.1038/s41598-023-33630-0

**Published:** 2023-04-20

**Authors:** Joel M. Garrett, Cedric Leduc, Zeki Akyildiz, Daniel J. van den Hoek, Filipe Manuel Clemente, Mehmet Yildiz, Hadi Nobari

**Affiliations:** 1 School of Health Sciences and Social Work, Griffith University, Southport, QLD Australia; 2 Carnegie Applied Rugby Research (CARR) Centre, Institute for Sport, Physical Activity and Leisure, Carnegie School of Sport, Leeds Beckett University, Leeds, UK; 3 Sport Science and Medicine Department, Crystal Palace FC, London, UK; 4 Training and Movement Science, Gazi University, Ankara, Turkey; 5 School of Health and Behavioural Sciences, University of the Sunshine Coast, Sippy Downs, QLD Australia; 6 Escola Superior Desporto e Lazer, Instituto Politécnico de Viana do Castelo, Rua Escola Industrial e Comercial de Nun’Álvares, 4900-347 Viana do Castelo, Portugal; 7 Instituto de Telecomunicações, Delegação da Covilhã, 1049‐001 Lisboa, Portugal; 8 Sports Science Faculty, Afyon Kocatepe University, Afyonkarahisar, Turkey; 9 Department of Exercise Physiology, Faculty of Educational Sciences and Psychology, University of Mohaghegh Ardabili, Ardabil, 56199-11367 Iran; 10 Faculty of Sport Sciences, University of Extremadura, 10003 Cáceres, Spain

**Keywords:** Physiology, Health care

## Abstract

This study examined the neuromuscular responses to three typical football (soccer) training sessions and the reliability of peak speed (PS) measured during a submaximal running test (SRT) for identifying neuromuscular fatigue (NMF) status. Jump height (CMJ_H_) and peak velocity (CMJ_PV_) were collected from a CMJ test, while peak speed (PS) was collected during an SRT before and after each training session. Large effect size (ES) decreases were observed in each variable post-training (ES; − 1.42 to − 2.32). Significant differences (> 0.001) were detected between each football session's external load variables. Coefficients of variations were small (< 10%) with moderate (CMJ_PV_; 0.53, PS; 0.44) and strong (CMJ_H_; 0.72) intraclass correlation coefficients between pre-test measures. The demands of each football session aligned with the principles of tactical periodization and were sufficient to produce the fatigue necessary to elicit physiological adaptations. PS was also shown to be a viable measure of monitoring NMF status.

## Introduction

In high-performance football (soccer), there is a growing popularity using the tactical periodization training model. This model aims to incorporate the game's tactical, technical, psychological, and physical qualities into training through a holistic approach^1^. The model aims for horizontal alternation, i.e., emphasising a given physical quality (e.g., strength, endurance, speed) in each session^2^. Splitting up the main physical qualities and focusing on each quality on a given day is thought to reduce any physiological interference from developing multiple qualities simultaneously^2,3^. This means the targeted training stimulus can be maximized while the other qualities recover, leading to greater adaptations^2,4^.

However, this model has received limited research despite its growing popularity^5^. The work that has been completed has shown that the model appears to be successful in differentiating training loads between specific training days^1,6^, but there is a lack of meaningful associations between specific metrics and changes in physical qualities^1,6^. For example, when monitoring this approach over a whole season, Lopstegui, Paulis and Escudero^6^ found that the workloads recorded in different football training sessions were significantly lower than the competition load for all the external and internal variables analysed (i.e. total distance, player load, distance above 14 km/h and 21 km/h etc.). While this maintained fitness levels across the season, it could not elicit any change in the physical qualities targeted.

To achieve optimal programming, it is essential to quantify a session's level of induced fatigue^2^. There is currently little known about the actual loading and neuromuscular impact of the typical sessions used within the tactical periodization training model^2^. Previous research suggests that training using the tactical periodization model may only induce movement-specific neuromuscular fatigue (NMF) responses^2^. While further research is needed to understand the neuromuscular impact of sessions used within the tactical periodization training model, the findings by Buchheit and colleagues^2^ support the growing evidence to suggest that the underlying mechanisms of fatigue are task-specific^7,8^. To assess the neuromuscular responses following team-sports sessions with, the countermovement jump (CMJ) test is commonly used owing to its robust reliability and validity^9,10^. However, team sports, such as football, appear to benefit from the analysis of the running profile to provide a greater task-specificity for monitoring NMF^2,8,11^. Monitoring the running profile, expressed as movement strategy changes, has been observed in practical standardised running tests^8,11,12^. However, the devices used in the current studies all contain accelerometers, which can be expensive in some sporting environments. Recently, Garrett et al.^13^ demonstrated that changes in the peak speed (PS) (observed as a reduction in the absolute value (km/hr) between pre- and post- session testing) measured during a submaximal running test (SRT) could provide an alternate method of measuring NMF when accelerometer devices are unavailable. Yet, there is currently a lack of information regarding the sensitivity of PS to different training sessions.

Therefore, the aims of the present study were to (1) examine the neuromuscular responses to three typical football training sessions used in the tactical periodization approach (strength, endurance and speed) in male semi-professional academy football players, and (2) examine the reliability of peak speed (PS) measured during a submaximal running test (SRT) compared to measures of a CMJ test (jump height and peak velocity).

## Methods

### Participants

This study used an observational, prospective cohort design and involved 20 male, Turkish, semi-professional, academy football players all aged over 18 years of age (age; 18.5 ± 0.5 years, weight; 68.6 ± 6.8 kg, height; 182.5 ± 4.3 cm). All 20 participants performed testing as part of their regular training regime and were familiar with procedures prior to the study. Informed consent was obtained from all participants, and the study was approved by the Afyon Kocatepe University Human Research Ethics Committee (Reference Number: 70306). This research was conducted following the ethical principles of the Declaration of Helsinki.

### Data collection

This study was performed in line with Buchheit and colleague's study^2^. The weekly training pattern completed was ‘strength’ on Tuesday, ' speed’ on Thursday in the first week, and ‘endurance’ on Wednesday in the second week. Data collection occurred during the “in-season” phase of the competion and was completed before and after each training session over the two-week training period for all participants (n = 20). Participants were included if they were a member of the Turkish, semi-professional, academy football program, free from injury, and provided written, informed consent.

### Data analysis

#### Countermovement jump test (CMJ)

As per established protocols^9^, CMJ testing was performed using an average of six CMJs for analysis. To align with previous research^8,12^ when using CMJ measures to monitor NMF for team sport athletes, CMJ height (CMJ_H_) and CMJ peak velocity (CMJ_PV_) were used as the main criterion measure of NMF. CMJ measures were obtained for analysis via an optical encoder (GymAware Power Tool, Kinetic Performance Technologies, Canberra, Australia) fixed to the ground and attached via a cable to the 400 g dowel rod. Like previous procedures^9^, subjects were encouraged to self-select the amplitude or rate of the countermovement with no attempts made to standardise these variables and keep the 400 g dowel rod positioned firmly across their shoulders in a horizontal plane. The validity and reliability of CMJ testing for NMF can be found elsewhere^8,9,14^.

#### Submaximal run test (SRT)

Following previously presented protocols by Garrett and colleagues^12,13^ and Leduc and colleagues^11^, the SRT involved four 50-m runs at five m/s with 20 s rest between trials. Participants were instructed to perform the run in 10 s with a time check at the 25-m halfway mark to help control for speed of the run. Athletes were familiarised with the requirements of the test (i.e. to complete the 50 m run in 10 s) and were instructed to run as near as possible to the 5 m/s target as possible. We recorded PS (km/h) throughout each of the four trials, with the average used as the criterion. Participants wore 10 Hz GPS units (Polar Team Pro Sensor, Kempele, Finland) between the scapulae in a specific tightly fitting vest. GPS signal reception was visually confirmed with the Polar software, requiring at least 4 satellites to display the athlete's unit number. Raw data files for each athlete were exported from Polar software (Polar Team Pro application, version 1.9.9, Polar Electro, Kempele, Finland) to visualize the collected variables with a timestamp. The validity and reliability of Polar Team Pro units can be found elsewhere^15–17^.

#### Football training sessions

Following the protocols by Buchheit and colleagues^2^, the three football training sessions were representative of three typical sessions in the tactical periodization training model (i.e., strength, endurance and speed, Table 1) targeting each of the three main physical qualities. While other coaches and/or organisations may choose different drills and exercises, it was decided to replicate the protocols by Buchheit and colleagues^2^ to compare findings in other populations. As in the study by Buchheit and colleagues^2^ the football training session with the highest level of neuromuscular demands (left column in Table 1) was referred to as the ‘strength’ session. The training load variables collected for each football session were: external load variables; total distance, meters per minute (m.min), total distance above 19.8 km h^−1^ (high-speed running (HSR), total distance above 25.2 km h^−1^ (very high-speed running (VHSR), PS; internal load variables; rating of perceived exertion (RPE).

**Table 1:** Football training sessions.

Strength	Endurance	Speed
1. Progressive plyometric drills (10 min)	1. Continuous 10–12-km/h run including whole-body mobility (12 min)	1. Running drills (10 min)
2. Strength stations (4 × 10-m lateral sprints with elastic bands, lateral lunges on a step + 5-m forward sprint, 6 single-leg forward hops + 5-m forward sprint, 5 + 5 + 5 + 5-m COD-sprint versus opponent, 4 × 15-m Power Sprints, 26 sprints—pulling equivalent of 24 kg26)	2. Technical warm-up (passing) (8 min)	2. Technical warm-up (passing) (5 min)
3. Technical warm-up (passing, 5 min)	3. Game with two small goals 4 versus 4 (40 × 35 m, three touches, 2 × 8 min, r = 90 s)	3. Possession 8 versus 8 (35 × 55 m, free touch, players need to receive the ball behind the goal line while not starting their run before the pass is initiated, 3 × 6 min, r = 90 s)
4. Game simulation 4 versus 4 + 2 goal keepers (width × depth, 30 × 25 m, three touches, 2 × 3 min, r = 90 s)	4. Same as 3 but goal only valid if all teammates have crossed the middle line	4. Sprint running (3 × 10 m, 3 × 15 m flying, 3 × 15 m standing start vs. opponent, 2 × 20 m standing start vs. opponent, r = 45 s)
5. Same as 4 but free touches and individual defence	5. Same as 4 but free touches + increased verbal encouragement from the coach	5. Same as 3 but increased verbal encouragement from the coach and increased emphasis on counter-attacking
6. Same as 4 but increased verbal encouragement from the coach		

### Statistical analysis

Statistical Analysis was completed in RStudio (2021.09.1 + 372 "Ghost Orchid" Release for macOS) for all analysis except for reliability, which was calculated using the spreadsheet for reliability by Hopkins^18^ (for review^9,19^). Descriptive statistics were computed for all variables from the CMJ test and SRT and reported as mean ± standard deviation (SD). Cohen’s d effect size (ES) statistic ± 95% confidence intervals (CI)) were calculated to determine the practical difference between the pre- and post-test for the CMJ and SRT variables. Differences represented as ES ± 95% CI were classified as; trivial (< 0.20), small (0.20–0.59), moderate (0.60–1.19), and large (1.20–1.99)^20^. The magnitude of the difference was considered ‘unclear’ where the 95% CI simultaneously overlapped the minor important ES (0.20) both positively and negatively^20^. Pearson’s correlations were calculated between each football training session's external and internal load variables and the change in NMF status. ANOVA post-hoc pairwise comparisons were conducted to determine the difference between training load variables from each football training session (i.e., strength vs speed, speed vs endurance etc.). The absolute and relative test–retest reliability for each variable was quantified via the typical error of measurements (TE) and expressed as a coefficient of variation (CV) ± 95% CI^18^. Intraclass correlations coefficients (ICC) were calculated for relative reliability between the football training sessions and pre-test measures. The correlations were classified as; very weak (0.00 to 0.19), weak (0.20 to 0.39), moderate (0.40 to 0.69), strong (0.70 to 0.89) and very strong (0.90 to 1.0)^21^. The smallest worthwhile change (SWC) was calculated as 0.20 × SD, representing a “small” effect size and a minor beneficial change in athletic performance^9^. Variables could detect the SWC if the TE ≤ SWC^10^.

## Results

**Figure 1:**
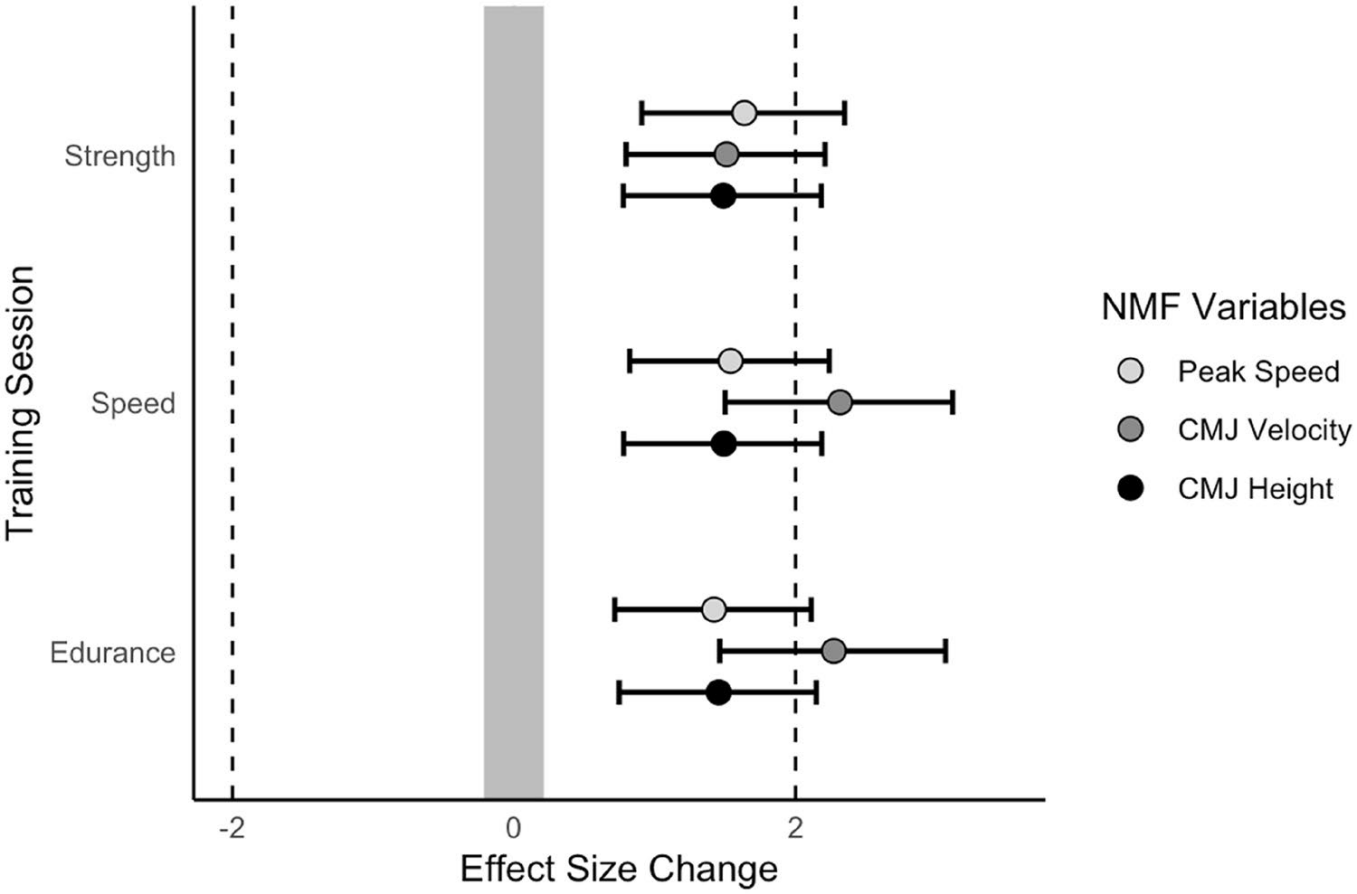
Effect size change between pre- and post-test neuromuscular fatigue variables. Grey = smallest important effect size (0.20) both positively and negatively. NMF; neuromuscular fatigue, CMJ; countermovement jump.

Descriptive statistics (mean ± SD) for each football training session are listed in Table 2. The differences between pre- and post-session NMF test variables for each session, represented as ES ± 95% CI, are described in Fig. 1. Compared to the pre-test measure for the ‘strength’ session (Table 1), a large decrease was observed for the post-test measure in CMJ_H_; ES − 1.49 (− 0.78 to − 2.18) and CMJ_PV_; ES − 1.51 (− 0.81 to − 2.21), and PS; ES − 1.64 (− 0.91 to − 2.35). Compared to the pre-test measure for the ‘endurance’ session, a large decrease in CMJ_H_; ES − 1.45 (− 0.75 to − 2.15) and CMJ_PV_; ES − 2.27 (− 1.46 to − 3.06), and PS; ES − 1.42 (− 0.71 to − 2.11) was observed post-session. Compared to the pre-test measure for the ‘speed’ session, a large decrease was observed for the post-test measure in CMJ_H_; ES − 1.49 (− 0.78 to − 2.19) and CMJ_PV_; ES − 2.32 (− 1.50 to − 3.11), and PS; ES − 1.54 (− 0.82 to − 2.24). For the ‘strength’ session, a moderate negative correlation (− 0.41) was observed between CMJ_PV_ and total distance and m.min, with a moderate positive correlation (0.41) observed between the change in PS and PS recorded within the strength session. For the ‘speed’ session, a moderate negative correlation (− 0.47) was observed between CMJ_PV_ and VHSR, and a moderate positive correlation (0.44) was observed between CMJ_H_ and VHSR distance. There were also significant differences (< 0.001) between the external load measures and the different football training sessions. However, there was no change between sessions for the internal load measure (RPE).

**Table 2:** Summary descriptive table (average (SD)) by groups of `football session type’.

	Strength	Endurance	Speed	Paired Comparison
	*N* = 20	*N* = 20	*N* = 20	
Duration (min)	85	105	80	N/A
Total Distance (m)	3559.95 (208.63)	7279.20 (310.96)	5218.80 (311.26)	All < 0.001
M.min^-1^	41.88 (2.45)	69.33 (2.96)	65.24 (3.89)	All < 0.001
HSR Distance (m)	32.65 (14.28)	61.05 (24.50)	340.05 (86.18)	All < 0.001
VHSR Distance (m)	0.00 (0.00)	3.00 (2.94)	340.00 (103.40)	All < 0.001
Peak Speed (km^-1^)	21.16 (0.98)	24.05 (1.39)	28.02 (2.16)	All < 0.001
RPE (A.U.)	6.30 (1.53)	5.90 (1.33)	5.50 (1.40)	strength-endurance; 0.65 strength-speed; 0.65 strength-endurance; 0.19

*N/A* not applicable, *A.U.* arbitrary units, *M min*^*−1*^ m per minute, *HSR* high-speed running > 19.8 km^−1^, *VHSR* very high-speed running > 25.2 km^−1^, *RPE* rating of perceived exertion.

Reliability statistics are shown in Table 3. Low absolute reliability was observed for all variables with CV’s of less than 10% (CMJ_H_; 4.7%, CMJ_PV_; 2.1, PS; 6.4) with moderate (CMJ_PV_; 0.53, PS; 0.44,) and strong (CMJ_H_; 0.72) ICCs. Only CMJ_H_ possessed a TE smaller than the SWC.

**Table 3:** Reliability of CMJ and SRT measures of neuromuscular fatigue.

	CMJ Height	CMJ Peak Velocity	SRT Peak Speed
	*N* = 60	*N* = 60	*N* = 60
Average (SD)	37.53 (3.61)	3.07 (0.29)	20.72 (1.48)
TE as %CV (± CI)	5.56 (4.52–7.76)	7.20 (5.76–9.91)	5.57 (4.46–7.65)
TE (± CI)	0.55 (0.44–0.75)	0.70 (0.57–0.96	0.76 (0.61–1.04)
ICC (± CI)	0.72 (0.48–0.87)	0.53 (0.22–0.76)	0.46 (0.12–0.71)
SWC	0.72	0.06	0.30

*CMJ* countermovement jump, *SRT* submaximal running test, *SD* standard deviation, *TE* typical error, *CI* confidence interval, *CV* coefficient of variation, *ICC* intraclass coefficient correlation, *SWC* smallest worthwhile change.

## Discussion

The results of the current study support those by Buchheit and colleagues^2^, with this sessions resulting in lower limb fatigue which may be associated with NMF. PS was also shown to be a viable measure of monitoring NMF status, supporting the previous literature^13^, that PS can offer an alternate task-specific method of measuring NMF when accelerometers are unavailable.

Considering we followed the protocols of Buchheit and colleagues^2^, it was unsurprising that the demands of each football training session (Table 2) were in line with the training prescription principles of tactical periodization, i.e., the emphasis on a given physical component in each different session. For instance, the ‘endurance’ session included a large activity volume (distance covered), while the ‘speed’ session showed greater distances of HSR activities. The training loads presented from the ‘strength’ session, however, may not accurately reflect the actual demands of that session. This is due to the lack of availability of accelerometers which meant we could not record mechanical load during the training sessions. The result is that highly demanding neuromuscular movements, such as weight pulling (i.e., power sprints) or plyometric drills, were not appropriately accounted for when analysing movement-based activity via non-accelerometer GPS units. Nonetheless, as in the study by Buchheit and colleagues^2^, the training contents (plyometric drills, high density small sided games) suggest that the physical objectives (i.e. exercises designed to elicit the highest level of neuromuscular demands) were likely matched for the ‘strength’ session.

The first finding of the present study is that the three football training sessions were all associated with a change in NMF status. Interestingly, this study saw large ES changes post-session, unlike those reported by Buchheit and colleagues^2^. There have been mixed responses reported in NMF status to team-sports sessions or game simulations^22,23^. The inconsistencies in the literature are likely due to differences in the study populations (i.e., age and individual characteristics), or these differences could be from testing in a real-life scenario. Testing in a practical setting could mean training-induced variations in players’ neuromuscular status between the different testing days due to additional days post-match and the influence of fatigue from a previous training session^2^. In this study, the players were semi-professional academy players who may not have the conditioning status to tolerate the same training sessions as the previously reported elite players. Nevertheless, if the goal is to develop various physical qualities, our results suggest the sessions delivered can be sufficient to generate fatigue important to elicit physiological adaptations. With the previous work^2^, these results provide additional information on how these typical training sessions can be manipulated to generate the desired training loads during different seasonal phases and in other athlete populations.

We observed conflicting results regarding the relationship between NMF variables and the training load variables. With the large ES changes observed pre- to post-session, there were expected to be greater relationships between training load variables and NMF measures, especially considering the significantly different external outputs between each training session. The moderate negative relationships between CMJ_PV_ and total distance and m.min aligns with similar findings that reported changes in NMF status due to increased collisions in Rugby League match play^24,25^. Increases in collisions were aligned with increases in accelerations and decelerations (mechanical load), often resulting in additional eccentric muscle damage that can manifest as a reduction in NMF status^25^. It is, therefore, plausible that the moderate negative correlation observed between CMJ_PV_ and the measures relating to distance could be the result of an increase in other variables, such as increased mechanical load during the ‘strength’ session. While not recorded in this study, by design, the ‘strength’ session included exercises of high neuromuscular demand (weight pulling, plyometric drills etc.) which would result in increases in accelerations and decelerations, additional eccentric muscle damage, and an increase in NMF. Subsequently, a moderate negative correlation was observed between CMJ_PV_ and VHSR distance for the ‘speed’ session. This negative correlation between CMJ_PV_ and VHSR is believed to be due to tissue damage evoked by the active lengthening of skeletal muscle common during HSR activities^25^. Evidence of this change in NMF status to HSR activities has been well documented within the literature^12,25–28^. The apparent sensitivity of CMJ_PV_ also aligns with the work of Garrett and colleagues^13^, who reported peak velocity recorded during a CMJ test as the most sensitive measure of NMF status out of the CMJ variables reported.

However, unexpectedly, we saw positive relationships between the change in PS, and PS recorded within the ‘strength’ session, and CMJ_H_ and VHSR distance during the ‘speed’ session. Some facilitation could explain this for muscle force application from better intramuscular coordination (i.e. greater synchronisation of motor units to increase force production)^2^. However, this remains unlikely with these variables' large ES changes post-session. Another explanation could be that different mechanisms influence these variables (CMJ_H_ and PS). It has been well-documented changes in NMF status can be the result of increases in accelerations, decelerations and HSR activities. However, the actual mechanism for this is yet to be known. The findings in this study need further investigation. Possibly including subjective measures of fatigue and/or muscle soreness or more intensive laboratory studies may be necessary to broaden our understanding of the specific mechanisms of NMF that different variables may be sensitive to^29^.

The small CVs and moderate to strong ICCs observed within the present study are comparable with previous findings^2,8,10,12^. However, only CMJ_H_ displayed a TE smaller than the SWC. The ability to define the magnitude of the SWC is complex and depends on several factors, such as the training context, the type of adaptations aimed to be monitored, and the observed variable itself^30^. Therefore, comparing the measures with thresholds for the magnitude of change is perhaps the best option for monitoring performance changes within individuals than relying solely on the SWC of test^30^. While it was expected that the ICC values to be higher in this study, these lower values could be attributed to what was mentioned above that during real-life scenarios, testing occurs within the training week, at different time points post-match and the influence of fatigue from a previous training session. This could decrease the reliability in results due to training-induced variations in players’ neuromuscular status between testing days.

Nonetheless, research on the reliability of CMJ or SRT measures for analysis of NMF supports their use in high-performance settings, and the current results of this study align with those reported previously^8–10,31,32^. Practically, this should give confidence to practitioners to utilise either method, but more specifically, certainty in the utility of PS as a viable measure of NMF status. Monitoring PS can enable practitioners to glean information about NMF status from a test administered as part of the warm-up, to a large group of athletes and be administered in under two minutes. It is also an inexpensive tool and can minimise the impact upon an athletes already busy schedule as it can be used to test within the normal training environment, such as the warm-up. As PS is a GPS metric, it enables real-time monitoring to inform immediate decisions on recovery status. This can allow timely decisions in multiple games per day and/or week and support decisions on rotations and recovery practices in the following games, trainings and recovery periods^13^.

This study was not without its limitations. With the study being underpowered, this could lead to the potential of underestimating the strength of evidence. Conflicting results were also reported between training load variables and measures of NMF status. The mechanisms causing NMF are still not fully known, and further analysis is needed to understand the sensitivity of each variable, the mechanisms behind the changes, and how they interact with different training load variables. For example, in a fatigued state, the difference in PS during the SRT appears to result from a more gradual acceleration, longer plateau run duration and a reduced deceleration at the end of standardised runs (see Fig. 2 in Garrett et al.^12^). The mechanism is thought to result from high stiffness increasing the stress induced by impact forces on the skeletal system^12^. The result is a change in movement pattern (observed by a reduction in PS) to reduce the stress on the skeletal system and minimise excessive muscle damage while maintaining performance output (i.e. time)^33^. However, this is an example of what has yet to be confirmed by the scientific literature and is currently only theorised, to identify some important areas for future research. As in previous research^12,13,34^, CMJ_H_ was also used as the main criterion measure of NMF, although research has shown an altered movement strategy (such as changes in PV) can be employed in the presence of NMF rather than just a reduced CMJ output^35^. The results of the current study, along with the previous literature^8,12–14,31,32^, confirms both jump height (CMJ_H_) and PV can be used as a sensitive measure of NMF. These results are in line with previous research analysing the sensitivity of monitoring NMF via a CMJ test as a comparison method with running profiles^34,36,37^, adding conficende to the current findings.

## Conclusion

In conclusion, the present results show that the demands of each football training session align with the principles of tactical periodization, i.e., the three typical football training sessions were representative of each of the three main physical qualities (strength, endurance, and speed). The large ES changes observed post-session suggests that horizontal alternation in programming is sufficient to generate fatigue and elicit physiological adaptations in this population. However, these results show that the typical football training sessions examined were not as well tolerated by semi-professional academy football players compared to what was reported for elite football players^2^. Additional support was also shown for GPS measures of PS to be a viable option for monitoring NMF status, providing an alternate task-specific method of measuring NMF when devices containing accelerometers are unavailable. Moreover, coaches should consider using changes in the average PS during the SRT to identify NMF and to adapt training programming accordingly.

## Data Availability

The data presented in this study are available on website: 10.6084/m9.figshare.21776828.v1 with Identifier:.
